# Proteomic Profiling of Outer Membrane Vesicles Released by *Escherichia coli* LPS Mutants Defective in Heptose Biosynthesis

**DOI:** 10.3390/jpm12081301

**Published:** 2022-08-09

**Authors:** Yaw-Kwan Chiu, Ti Yin, Yi-Tzu Lee, Shyi-Jou Chen, Yung-Chih Wang, Kuo-Hsing Ma

**Affiliations:** 1 Graduate Institute of Medical Sciences, National Defense Medical Center, Taipei 114, Taiwan; 2 Department of Pediatrics, Songshan Branch, Tri-Service General Hospital, National Defense Medical Center, Taipei 105, Taiwan; 3 School of Nursing, National Defense Medical Center, Taipei 114, Taiwan; 4 Department of Nursing, Tri-Service General Hospital, National Defense Medical Center, Taipei 114, Taiwan; 5 Department of Emergency Medicine, Taipei Veterans General Hospital, Taipei 112, Taiwan; 6 Faculty of Medicine, School of Medicine, National Yang-Ming University, Taipei 112, Taiwan; 7 Department of Pediatrics, Tri-Service General Hospital, National Defense Medical Center, Taipei 114, Taiwan; 8 Division of Infectious Diseases and Tropical Medicine, Department of Internal Medicine, Tri-Service General Hospital, National Defense Medical Center, Taipei 114, Taiwan; 9 Department of Biology and Anatomy, National Defense Medical Center, Taipei 114, Taiwan

**Keywords:** *E. coli*, iTRAQ, LPS, OMV, *rfaC*, *rfaG*, rfaL, wound

## Abstract

*Escherichia coli* releases outer membrane vesicles (OMVs) into the extracellular environment. OMVs, which contain the outer membrane protein, lipopolysaccharides (LPS), and genetic material, play an important role in immune response modulation. An isobaric tag for relative and absolute quantitation (iTRAQ) analysis was used to investigate OMV constituent proteins and their functions in burn trauma. OMV sizes ranged from 50 to 200 nm. Proteomics and Gene Ontology analysis revealed that Δ*rfaC* and Δ*rfaG* were likely involved in the upregulation of the structural constituent of ribosomes for the outer membrane and of proteins involved in protein binding and OMV synthesis. Δ*rfaL* was likely implicated in the downregulation of the structural constituent of the ribosome, translation, and cytosolic large ribosomal subunit. Kyoto Encyclopedia of Genes and Genomes analysis indicated that Δ*rfaC* and Δ*rfaG* downregulated *ACP*, *ACEF*, and *ADHE* genes; Δ*rfaL* upregulated *ACP, ACEF*, and *ADHE* genes. Heat map analysis demonstrated upregulation of *galF, clpX, accA, fabB*, and *grpE* and downregulation of *pspA, ydiY, rpsT*, and *rpmB*. These results suggest that RfaC, RfaG, and RfaL proteins were involved in outer membrane and LPS synthesis. Therefore, direct contact between wounds and LPS may lead to apoptosis, reduction in local cell proliferation, and delayed wound healing.

## 1. Introduction

**Figure 1:**
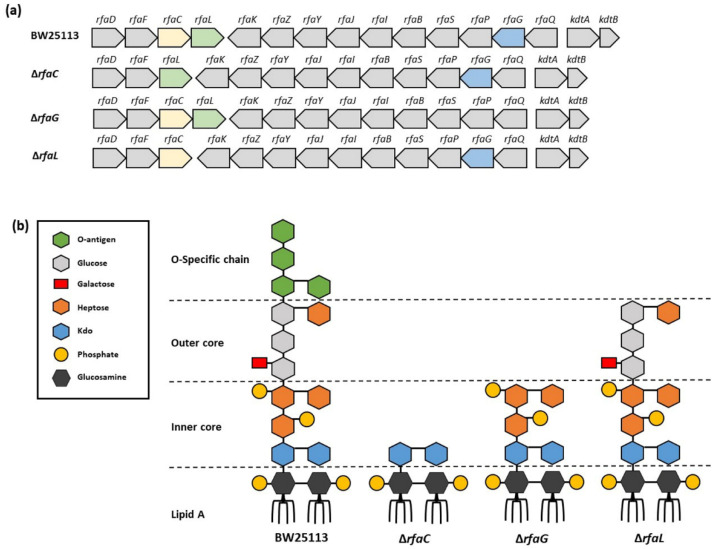
Lipopolysaccharide (LPS) architecture of OMVs. (**a**) OMV gene organization of *E. coli* BW25113, *rfaC* knockout (Δ*rfaC*), *rfaG* knockout (Δ*rfaG*), and *rfaL* knockout (Δ*rfaL*) strains, respectively; (**b**) LPS architecture of OMVs of *E. coli* BW25113, *rfaC* knockout (Δ*rfaC*), *rfaG* knockout (Δ*rfaG*), and *rfaL* knockout (Δ*rfaL*) strains, respectively. This figure is adapted from Pragnout et al. [17] and is licensed under a Creative Commons Attribution 4.0 International License (http://creativecommons.org/licenses/by/4.0/, accessed on 28 December 2021).

Gram-negative bacteria release nanovesicles from their outer membrane into the extracellular milieu which are called outer membrane vesicles (OMVs) [1,2]. OMVs, which range from 20 to 200 nm in size, have a lipid-bilayered diameter and spherical proteolipids. They contain lipopolysaccharides (LPS), periplasmic proteins, outer membrane lipids, cytoplasmic proteins, DNA, RNA, outer membrane proteins, and other elements related to virulence [3,4,5,6,7]. OMVs play important roles in bacterial physiological and phthological aspects such as genetic material transfer, protein transport, nutrient acquisition, antibacterial activity, virulence factor delivery, interkingdom communication, immune response modulation, and neutralizing phage decoy activity [8,9,10,11,12,13,14]. As the major component of OMV, LPS contains lipid A, a core oligosaccharide, and the O-specific polysaccharide of the O-antigen (Figure 1b) [15]. LPS has been reported to be associated with infection. In burn injury and infection-related diseases, LPS of pathogens interact with membrane-bound or soluble CD14, lipopolysaccharide-binding protein (LBP), and Toll-like receptor 4 (TLR4) to initiate cellular production of pro-inflammatory cytokines, immune cell recruitment, and endotoxin clearance [15]. In *Escherichia coli*, the synthesis of LPS required *rfa* (also known as *waa*) operons that consist of many genes [16]. Three operons regulate the genes in the *rfa* locus. The first operon comprises *rfaC (waaC), rfaD* (or *gmhD*), *rfaF* (or *waaF*)*,* and *rfaL* (or *waaL*) genes. Then, *rfaB* (or *waaB*)*, rfaG* (or *waaG*), *rfaI* (or *waaO*), *rfaJ* (or *warJ/waaJ*), *rfaK* (or *waaU*), *rfaP* (or *waaP*)*, rfaQ* (or *waaQ*)*, rfaS* (or *waaS*)*, rfaY* (or *waaY*), and *rfaZ* (or *waaZ*) are organized in the second operon. The remaining short *kdtA* operon consists of *kdtA* (or *waaA*) and *kdtB* (or *coaD*) [17,18,19,20]. In the LPS core biosynthesis pathway, rfaC has been proposed to play a crucial role in transferring heptose to the LPS core.

The destruction of the LPS structure would influence the microbiological features of the bacteria and result in the alternation of the OMVs they released. Nakao et al. have reported that mutation in *rfaC* produced defective LPS in OMV but still maintained membrane integrity. *E. coli* with *rfaC* mutation generated OMV comparable to the wild type but produced more extracellular DNA (eDNA) in the culture, which is involved in initial attachment and biofilm formation as well as enhancing cell wall hydrophobicity [21,22]. Upon formation in the wound site, biofilms can have harmful effects including impaired epithelialization, granulation tissue formation, and reduced inflammatory response, which delays the healing process [23,24]. Several studies also mentioned that mutations in the *rfa* locus lead to severely truncated LPS and result in elevated antibiotic resistance, multidrug resistance, and resistance to several bacteriophages used in therapy for bacterial infections [25]. In burn injury patients, this phenomenon delays the wound healing process [26]. Another study reported that glycosyltransferase activity and O-antigen attachment to lipid A in *E. coli* was affected by mutations in the *rfaL* gene, thereby reducing biofilm formation [27]. Owing to the highly conserved inner-core composition of Gram-negative bacteria, the *rfa* gene may be a possible therapeutic target against infection.

Proteomic analysis of the entire set of proteins has been used to specify the composition of OMVs in several studies [1,15]. However, these studies remain limited, and only a few proteins have been recognized [6,7]. The main objective of this study was to elucidate the effect of LPS structure on OMV composition using the proteomics analysis. Based on the characteristics of the OMV genes above, we profiled *rfaC, rfaG,* and *rfaL* using quantitative proteomics to investigate *E. coli* outer membrane genes, and interpreted the effect of LPS structure on OMV composition. The novelty of this study is to provide proteomic data of the truncated RfaC, RfaG, and RfaL proteins in *E. coli* to support claims in previous publications that Rfa proteins could be a target for the treatment of the infected wounds of patients. An isobaric tag for relative and absolute quantitation (iTRAQ)-based proteomic analysis technique was employed to identify the expressed proteins between OMVs released by *E. coli* BW25113 and its mutant strains, including the *rfaC-*, *rfaG-*, and *rfaL*-defect strains. The functional classification of proteins and key pathways were analyzed by utilizing the Gene Ontology (GO) annotation and Kyoto Encyclopedia of Genes and Genomes (KEGG) pathway enrichment analysis, respectively. Our results provide essential information regarding the mechanism of the expressed genes and key pathways among proteins and *E. coli* strains.

## 2. Materials and Methods

### 2.1. Bacterial Strains, Cultures, and Growth Conditions

*E. coli* strain BW25113 and its mutants with deleted rfaC, rfaG, and rfaL genes were used (Figure 1, Table 1). All strains were purchased from Horizon Discovery (Cambridge, UK). The *E. coli* BW25113 strain is a K-12 derivative with a recA+ and hsdR genotype and the parent of the Keio collection of single-gene knockouts which has >100-fold higher transformation efficiency than the commonly used cloning *E. coli* hosts. The rfa genes in the mutant strains were deleted by using a one-step gene deletion method with the phage lambda Red recombinase. Bacteria were maintained and grown on agar at 37 °C with aeration.

### 2.2. OMV Sample Preparation

OMVs were isolated from the late log-phase (16 h) culture of *E. coli*. In brief, 200 mL LB was inoculated with 2 mL overnight culture medium, incubated at 37 °C, and agitated by shaking at 200 rpm for 6 h. The cells were pelleted by centrifugation (10,000 rpm for 15 min) and the supernatant was filtered through a 0.22 μm membrane filter (JET BIOFIL, Guangzhou, China) to remove cells and cellular debris. The filtrate was subjected to ultracentrifugation (35,000 rpm) for 2 h at 4 °C using a Type 45 Ti rotor (Beckman, CA, USA). For washing the OMVs, the pellet was suspended in phosphate-buffered saline (PBS) and then ultracentrifuged (42,000 rpm) for 2 h at 4 °C using the same rotor. The pellet was finally resuspended in PBS and stored on ice. Three biological replicates of each experiment were performed in the following analysis.

### 2.3. Transmission Electron Microscopy (TEM)

Vesicles that were obtained were placed on a glow-discharged carbon-coated EM grid and allowed to rest briefly. They were then gently washed with deionized water twice and the grids were stained with 1% uranyl formate and dried at room temperature overnight. The grids were then observed using a transmission electron microscope (FEI Tecnai G2 F20 S-TWIN, FEI Company, Hillsboro, OR, USA) at 120 kV. TEM figures were visualized using the ImageJ software (National Institutes of Health, Bethesda, MD, USA) and analyzed using Student’s *t*-test. Diagrams were generated using the GraphPad Prism software (version 8.0, GraphPad software, San Diego, CA, USA) [28,29].

### 2.4. Nanoparticle Tracking Analysis (NTA) of OMVs

The quantities and sizes of OMVs were measured using the NanoSight NS500 nanoparticle tracking system (NTA) (Malvern Instruments Ltd., Malvern, Worcestershire, UK) supplied with a 488 nm blue laser and camera with a complementary metal-oxide semiconductor (CMOS) image sensor. Frozen OMV samples were thawed at room temperature and diluted 1/1000 in 50 mM HEPES buffer at pH 7.4 before analysis. Polystyrene beads (100 nm diameter) were used as the positive control and HEPES buffer alone served as the negative control. Samples were pumped into a NanoSight instrument using a syringe and set at the ‘20′ speed setting (in arbitrary units) on the NS500. The quantification was captured in five 60 s reads at room temperature (at approximately 23.9–25.2 °C), and the instrument was optimized at automatic setting (for ‘blur,’ minimum track length,’ and ‘minimum expected size’ setting), whereas viscosity was set to ‘water’ (0.883–0.911 cP). Camera level and focus for automated image setup were chosen for video enhancement, and the NTA software version 2.3 (Malvern Instruments Ltd., Malvern, Worcestershire, UK) was used to determine a total of 1498 frames per sample with a threshold of 5 (in arbitrary units). The data, including mean size (nm), mode size (nm), and concentration (particles/mL), were arranged, and an average of five reads was measured and plotted as particle size versus number of particles per Ml using Origin85 and the average size of OMVs was calculated using GraphPad Prism software [30].

### 2.5. Protein Extraction

The *E. coli* cultures were incubated at 37 °C for 16 h to acquire the total protein of OMVs. The cultures were centrifugated at 5000× g at 4 °C for 10 min, followed by protein extraction as previously described [24]. Trypsin digestion was then performed for 100 g of total cellular protein from each sample using Trypsin Gold (Promega, Madison, WI, USA) at a mass ratio of 30:1 trypsin-to-protein extract. Reactions were incubated at 37 °C for 16 h. Afterward, the digested proteins were dried using vacuum centrifugation [31].

### 2.6. iTRAQ Labeling and SCX Fractionation

First, 0.5 M triethylammonium bicarbonate (TEAB) was used to dissolve the dried peptide samples. Then, the peptide samples from *E. coli* cultures were labeled with iTRAQ reagents 114 and 116, respectively. The labeled proteins were incubated at room temperature for 2 h, then collected and dried using vacuum centrifugation. The protein labeled by iTRAQ was dissolved in 4 mL buffer A (25 mM NaH_2_PO_4_ in 25% ACN, pH 2.7) and separated using an LC-20AB HPLC pump system (Shimadzu, Kyoto, Japan) with Ultramex SCX column (4.6 mm × 250 mm, 5 µm, Phenomenex, Torrance, CA, USA). The sample was eluted by the linear gradient of buffer A for 10 min, 50–60% buffer B (25 mM NaH_2_PO_4_ and 1 M KCl in 25% ACN, pH 2.7) for 27 min, and the 60–100% buffer B for 1 min. Subsequently, the peptide was eluted every minute to 20 fractions at a flow rate of 1 mL/min and an absorbance wavelength of 214 nm. Finally, the protein fractions were desalted using Strata X C18 (Phenomenex, Torrance, CA, USA) and dried using vacuum centrifugation [32].

### 2.7. LC–MS/MS Analysis

The labeled protein fractions that were diluted in 40 µL 0.1% (*v/v*) trifluoroacetic acid were introduced to a nanoLC–MS/MS for analysis (Q Exactive mass spectrometer, Thermo Fisher Scientific, Waltham, MA, USA), performed in positive ion mode coupled with Easy nLC (Thermo Fisher Scientific, Waltham, MA, USA) for 60 min. MS data were obtained using a data-independent top 10 method. Furthermore, automatic gain control (ACG) target was set to 1e6 (1e6 = 1,000,000), the maximum inject time to 50 ms, and the duration of Dynamic exclusion was 60 s. Survey scans were obtained at a resolution of 70,000 and *m/z* 200, and isolation width was 2 *m/z*. Normalized collision energy was 30 eV, and underfill ratio, which determines the minimum percentage of the target value possible to be gained at the maximum fill time, was determined as 0.1%. The instrument was performed with peptide identification mode permitted [33].

### 2.8. Proteomic and Bioinformatic Analysis

Protein characterization was conducted using Mascot^®^ (version 2.2; Matrix Science, MA, USA) and the Proteome Discoverer™ software (version 1.4; Thermo Scientific, Waltham, MA, USA) using the sequences from the UniProt Human Database (133,549 sequences, downloaded on 3 March 2013). In this method, the parameters used were mass tolerance = 20 ppm, MS/MS tolerance = 0.1 Da, enzyme = trypsin, missed cleavage—2, oxidation (M), iTRAQ 8plex (Y) as the possible variable modifications, and carbomidomethyl (C), iTRAQ 8plex (N-term), iTRAQ 8plex (K) as the permanent modifications. The calculation for false discovery rate (FDR) of peptide characterization used a bait database search at a filtering basis of FDR ≤0.01. The iTRAQ ratio between the two groups of >1.2 or <0.83 defined the differential protein expression, and all of the diversely expressed RfaC, RfaG, and RfaL proteins were examined using UniProt (http://www.uniprot.org/; accessed on 28 December 2021) [34].

A Venn diagram was generated to elaborate the general diversely expressed proteins between the OMVs released by *E. coli* wild-type (BW25113) and its mutant strains. The cross-comparison of the gene names generated Venn diagrams and sets of gene lists are shown in Table 2. GO analysis (version go_201608.obo; www.geneontology.org; accessed on 28 December 2021) was used to examine the biological importance of the distinct expressed proteins. Furthermore, the distinct expressed proteins were entangled in the identical process; function and components were distributed into corresponding clusters. KEGG pathway analysis was carried out to explore the potential of the biological pathways using the online software (KEGG Automatic Annotation Server (KAAAS)) [33].

### 2.9. Statistical Analysis

All data were statistically analyzed using IBM^®^ SPSS^®^ Software Version 18.0 (IBM Corp., Armonk, NY, USA) and expressed as mean ± standard error. Data were analyzed using one-way analysis of variance or a two-tailed paired *t*-test. Significant differences between groups were detected using * *p* < 0.05 and ** *p* < 0.01 to indicate statistical significance.

## 3. Results

### 3.1. TEM Analysis of OMVs Released from E. Coli BW25113 and Mutants

**Figure 2:**
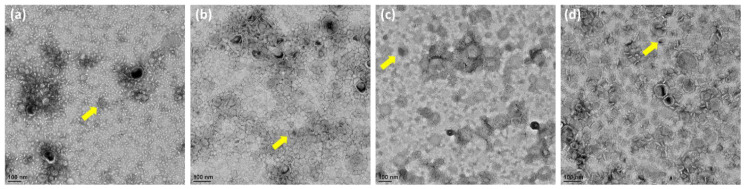
Morphology and size of OMVs from WT and mutant *E. coli*. (**a**–**d**) Transmission electron microscopy observation of *E. coli* BW25113, rfaC knockout (ΔrfaC), rfaG knockout (ΔrfaG), and rfaL knockout (ΔrfaL) strains, respectively. The yellow arrows show the example of observed OMV.

Generally, in Figure 2a–d, OMVs were not uniform in size. High magnification TEM images of OMVs showed spherical particles. However, the overall sample quantification data showed that the size of the OMVs released by the Δ*rfaG* strain was significantly larger than that of *E. coli* BW25113, whereas OMVs released by the Δ*rfaC* and Δ*rfaL* strains were significantly smaller than that of *E. coli* BW25113.

### 3.2. NTA Analysis of OMVs from WT and Mutant E. Coli

**Figure 3:**
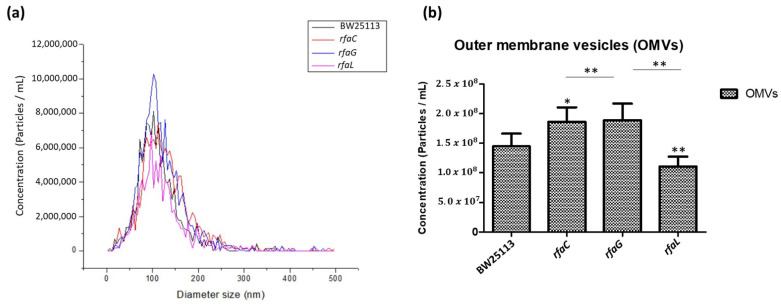
Small and large outer membrane vesicle (OMV) populations purified from a heterogeneous population of OMVs. (**a**) OMV population diameter sizes (nm) of *E. coli* BW25113, *rfaC* knockout (Δ*rfaC*), *rfaG* knockout (Δ*rfaG*), and *rfaL* knockout (Δ*rfaL*) strains, respectively, were evaluated using Nanoparticle Tracking Analysis (NTA); (**b**) average diameter size of *E. coli* BW25113, *rfaC* knockout (Δ*rfaC*), *rfaG* knockout (Δ*rfaG*), and *rfaL* knockout (Δ*rfaL*) OMVs presented in nm in each concentration (particle/mL). * *p* < 0.05 and ** *p* < 0.01 indicate statistical significance.

To further determine the distribution of OMV size released by the *E. coli* BW25113 and its mutant strains, we analyzed the heterogenous population of OMVs using NTA. The results revealed that the size distribution of OMVs in each group ranged from 50 to 200 nm in diameter, but the majority were 100 nm (Figure 3a). Furthermore, the histogram data showed that OMVs released by the Δ*rfaC* and Δ*rfaG* strains were significantly larger in diameter compared with those of the Δ*rfaL* strain (Figure 3b).

### 3.3. Proteomic Analysis of the OMV Fraction from E. Coli

**Figure 4:**
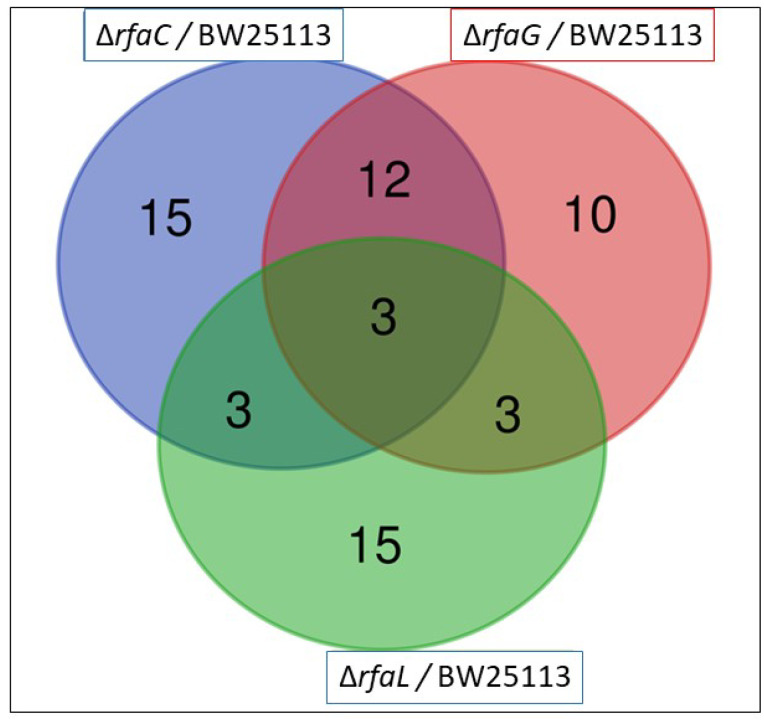
Venn diagram of the existence of the OMV genes in *E. coli* BW25113, *rfaC* knockout (ΔrfaC), *rfaG* knockout (Δ*rfaG*), and *rfaL* knockout (Δ*rfaC*) strains. Three strains encompassing *rpsT*, *galF*, and *rpmB* existed in the three *E. coli* mutants.

The OMV pan-proteome was determined after the identification of clusters of orthologous groups (COG) (Figure 4 and Table 2). Individual cross-comparison of shared genes from Venn diagrams are summarized in Table 2. The identified proteins were grouped into 33, 28, and 24 clusters of orthologous genes for OMVs purified from *E. coli* BW25113 and mutant strains (Δ*rfaC*, Δ*rfaG*, and *ΔrfaL* genes, respectively) (Figure 4). Only 3 clusters containing RpsT, GalF, and RpmB proteins were shared among all the strains (Table 2). The RpsT protein, which defines the 30S ribosomal protein S20 OS, was downregulated in *E. coli* mutants (Δ*rfaC* log2 ratio = −2.124; Δ*rfaC* normalized ratio = 0.229; Δ*rfaG* log2 ratio = −1.986; Δ*rfaG* normalized ratio = 0.253; Δ*rfaL* log2 ratio = −1.968; and Δ*rfaC* normalized ratio = 0.256). The 50S ribosomal protein L28 OS, defined by the RpmB protein, was also downregulated in all mutants (Δ*rfaC* log2 ratio = −2.181; Δ*rfaC* normalized ratio = 0.220; Δ*rfaG* log2 ratio = −3.315; Δ*rfaG* normalized ratio = 0.100; Δ*rfaL* log2 ratio = −2.844; and Δ*rfaL* normalized ratio = 0.139). Further, the GalF protein upregulated UTP-glucose−1-phosphate uridylyl transferase OS in *E. coli* BW25113 mutants (Δ*rfaC* log2 ratio = 1.792; Δ*rfaC* normalized ratio = 3.463; Δ*rfaG* log2 ratio = 1.588; Δ*rfaC* normalized ratio = 3.006; Δ*rfaL* log2 ratio = 1.317; and Δ*rfaL* normalized ratio = 2.492). Venn diagrams showing shared and unique genes in *E. coli* BW25113, *rfaC* knockout (*ΔrfaC*), *rfaG* knockout (Δ*rfaG*), and *rfaL* knockout (Δ*rfaC*) strains were *rpsT*, *galF*, and *rpmB*, respectively. The log2 fold values were shown in Table 3.

### 3.4. Heat Map Analysis of Mutant Strains Compared to E. Coli BW25113 Strain

**Figure 5:**
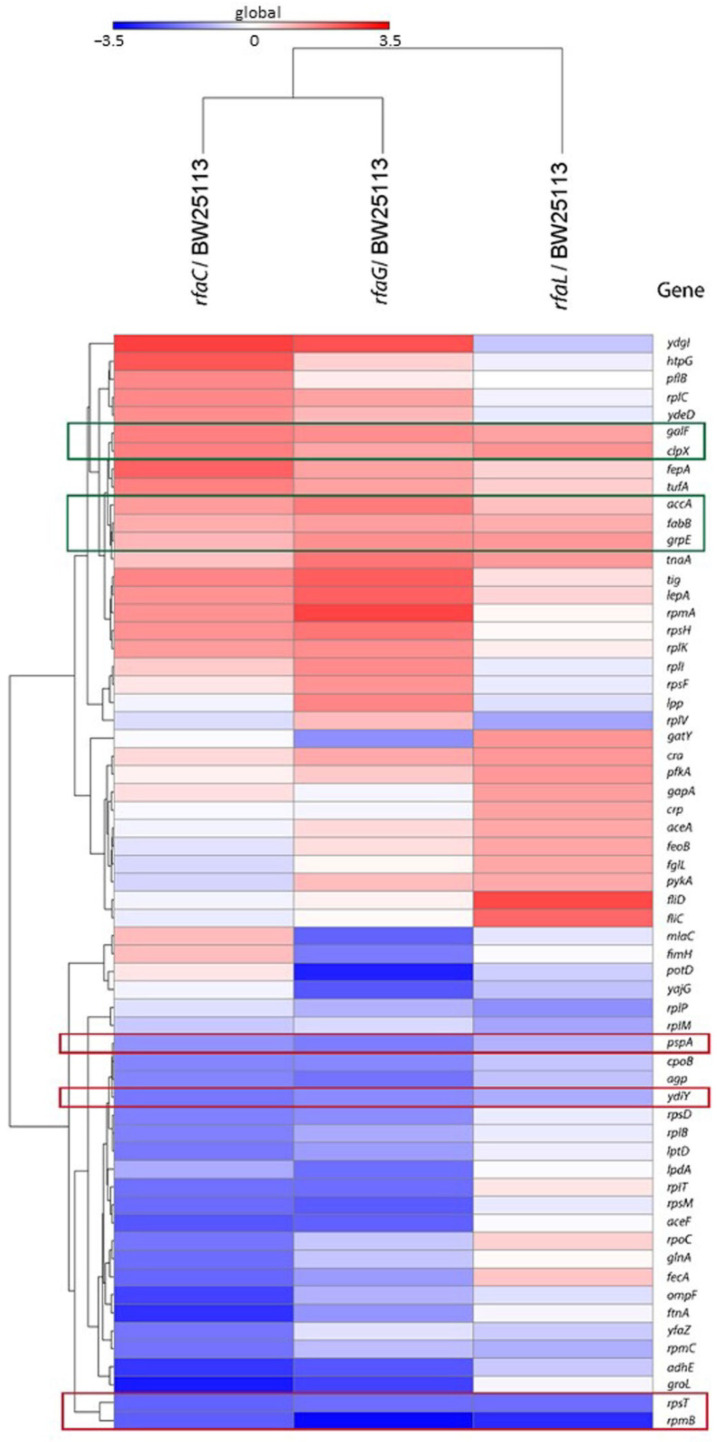
Heat map analysis of *E. coli* BW25113 mutant strains (*rfaC* knockout (Δ*rfaC*), *rfaG* knockout (Δ*rfaG*), and *rfaL* knockout (Δ*rfaL*) strains) genes shared with WT *E. coli* BW25113 strain. Red frames are for downregulated genes, whereas the green frames are the upregulated genes.

Heat map analysis showed that GalF, ClpX, AccA, FabB, and GrpE proteins were upregulated among the OMVs released by the mutant strains compared with the *E. coli* BW25113 (Figure 5). In contrast, PspA, YdiY, RpsT, and RpmB proteins were found to be downregulated in mutant strains compared with *E. coli* BW25113.

### 3.5. GO Bar Chart of Mutants Versus E. Coli BW25113 strains

**Figure 6:**
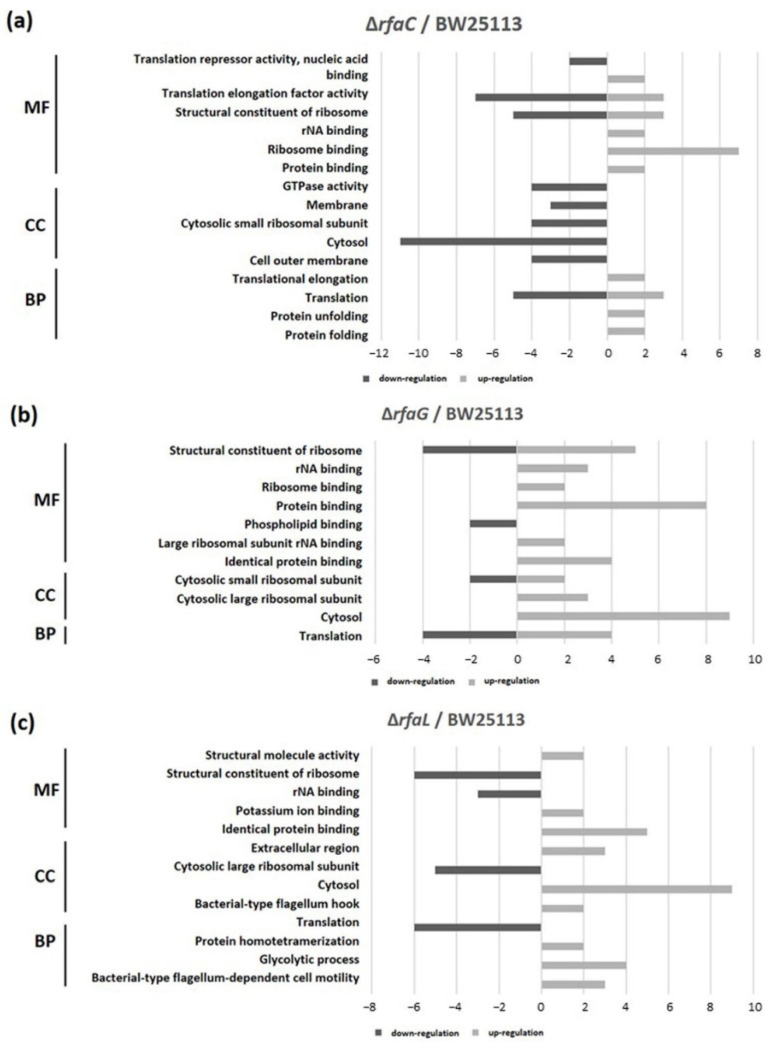
Functional classification of OMV proteins. Gene ontology (GO) classification was conducted to determine protein content. (**a**) *E. coli* BW25113 Δ*rfaC* strain and *E. coli* BW25113 shared clusters. (**b**) *E. coli* BW25113 Δ*rfaG* strain and *E. coli* BW25113 shared clusters. (**c**) *E. coli* BW25113 Δ*rfaL* strain and *E. coli* BW25113 shared clusters. MF: Molecular Function; CC: Cellular Component; BP: Biological Process.

GO classification revealed that protein binding was the most shared in the OMVs of Δ*rfaC* and that of BW25113 strain, whereas structural constituent of ribosome and rRNA binding were the least shared in molecular function (MF). Cytosol and translation clusters of the Δ*rfaC* strain were downregulated in the cellular component (CC) and biological process (BP), respectively (Figure 6a). Between Δ*rfaG* and BW25113 strains, protein binding and structural constituent of ribosome were upregulated in MF, and cytosol was abundant in CC (Figure 6b). Additionally, for the Δ*rfaL* strain versus wild-type BW25113, cytosol and identical protein binding were the most prevalent in CC and MF, respectively. In addition, the structural constituent of ribosome, translation, and cytosolic large ribosomal subunit were strongly downregulated between Δ*rfaL* and BW25113 strains (Figure 6c).

### 3.6. Pathways Predicted by KEGG

**Figure 7:**
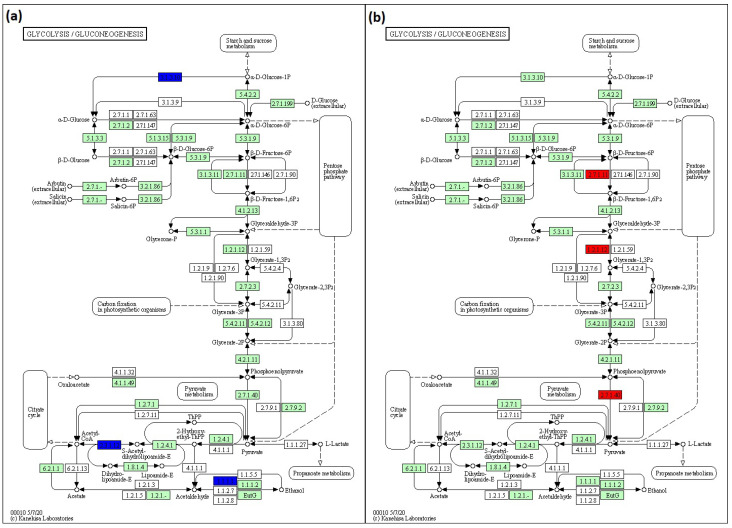
KEGG pathway maps for predicting pathways influenced by the lack of (**a**) *rfaC* and *rfaG*, and (**b**) *rfaL* OMV genes in *E. coli* BW25113. This pathway is produced using KEGG Mapper online software (https://www.genome.jp/kegg/mapper/color.html; accessed on 28 December 2021).

To predict pathways, KEGG analysis was conducted. In glycolysis/glucogenesis, lack of *rfaC* and *rfaG* genes in *E. coli* BW25113 was found to downregulate acyl carrier protein (*ACP),* acetyltransferase component of pyruvate dehydrogenase *(ACEF*), and aldehyde-alcohol dehydrogenase (*ADHE*) genes. The downregulation pathways in BW25113 mutants influenced by the lack of *rfaC* and *rfaG* genes were depicted in KEGG pathway maps as 3.1.3.10, 2.3.1.12, and 1.1.1.1 for *ACP*, *ACEF*, and *ADHE*, respectively (Figure 7a). The normalized ratios of the *rfaC* gene versus BW25113 were 0.319, 0.205, and 0.152 for *ACP*, *ACEF*, and *ADHE*, respectively. The normalized ratios of the *rfaG* gene lacking from BW25113 were 0.270, 0.227, and 0.207 for *ACP*, *ACEF*, and *ADHE*, respectively. In addition, the lack of the *rfaL* gene upregulated phosphofructokinase isozyme (PFKA)*,* glyceraldehyde-3-phosphate dehydrogenase-A (GAPA), and pyruvate kinase isozyme (PYKA) in the BW25113 mutant in glycolysis/glucogenesis. The items in the KEGG pathway map displayed the abundance of those pathways using 2.7.1.11, 1.2.1.12, and 2.7.1.40 map items (Figure 7b). A deleted *rfaL* gene resulted in 2.721, 2.589, and 2.313 normalized ratios for PFKA*,* GAPA, and PYKA, respectively.

## 4. Discussion

Vesicles obtained from the outer membrane of Gram-negative bacteria are known as OMVs and are highly diverse in size, composition, and function [35]. In this study, TEM was used to determine the appearance and size of OMVs released by *E. coli* BW25113 and *rfaC, rfaG,* and *rfaL* knockout strains (Figure 2a–d). Moreover, using NTA, the diameter of OMVs in all strains was shown to range from 50–200 nm (Figure 2a), with the highest mostly found in *E. coli* lacking *rfaC,* and *rfaG*, significantly different from OMVs released by *E. coli* BW25113 and *E. coli* lacking *rfaL* (Figure 2b). In addition, our NTA results showed that OMVs released by *E. coli* BW25113 and its mutant strains ranged between 50–200 nm in diameter, with the majority being 100 nm (Figure 3a). Additionally, the OMVs of Δ*rfaC* and Δ*rfaG* were found to be larger than those of BW25113 and Δ*rfaL* (Figure 3b). These results indicate that secreted OMVs have diverse functions. Turner et al. mentioned that OMV size is dependent on protein content and composition [30]. The smaller OMV fraction contained less protein than the larger OMV fraction and the heterogenous population of OMVs. Generally, the larger OMVs contained more adhesion protein for virulence, whereas small OMVs contained protein predominantly for metabolism. The size of OMVs also determined the mechanism and efficiency of cellular entry of the OMV into the host cell. Smaller OMVs are known to enter the cell through the micropinocytosis pathway, whereas larger OMVs enter the cell preferentially via the clathrin and dynamin-mediated pathway. Inhibition of clathrin-mediated endocytosis has no effect on enterotoxigenicity of *E. coli* OMVs [30,36].

Furthermore, in the proteomics study, the genes *rpsT, galF*, and *rpmB* were identified as shared among cluster mutants and *E. coli* BW25113 (Figure 4). Our results revealed that *rpsT*, which defines the 30S ribosomal protein S20 OS, and 50S ribosomal protein L28 OS that is encoded by the *rpmB* gene, were downregulated in both mutant and *E. coli* BW25113 strains. On the other hand, *galF* upregulated UTP-glucose-1-phosphate uridylyl transferase OS in *E. coli* BW25113 mutants. These proteomic data were consistent with the heat map analysis *results. GalF, ClpX, AccA, FabB, and GrpE appeared* to be upregulated among all OMV proteins in the *E. coli* BW25113 and mutant strains (Figure 5). This result likely suggests that *rfaC*, *rfaG*, and *rfaL* are not directly responsible for fatty acid biosynthesis and elongation together with AccA and FabB, respectively [37,38]. Furthermore, GalF proteins for cellular UDP-glucose formation and GrpE for protein folding and thermos-sensing demonstrate a high protein expression following OMV protein knockout (Δ*rfaC*, Δ*rfaG*, Δ*rfaL*) in mutant and *E. coli* BW25113 strains, indicating that UDP-glucose formation and heat shock response genes continue to function well without the presence of OMV proteins [39,40]. However, the downregulation of stress-related proteins such as PspA and YdiY [41,42] is likely attributable to their relationship with OMV protein construction, and the suppression of the aforementioned gene transcripts or protein expression by OMV protein knockout. Moreover, upregulation of the ClpX protein and downregulation of ribosomal proteins such as RpsT and RpmB are suggested to occur because of the involvement of these OMV genes in ribosomal 30S and 50S synthesis, respectively [43,44].

The results of GO exhibited that the binding protein is the most widely shared protein in Δ*rfaC* and *E. coli* BW25113 strains, whereas the structural constituent of the ribosome and rRNA binding are the least conserved. Moreover, cytosol and translation clusters of Δ*rfaC* in CC and BP, respectively are downregulated (Figure 6a). These data are reasonable considering the importance of OMVs in supporting the unique architectures corresponding to protein transport, genetic material transfer, interkingdom communication, antibacterial activity, neutralizing phage decoy activity, virulence factor delivery, and immune response modulation that involve binding activity [8,9,10,11,12,13,14]. Furthermore, the expression of protein binding and structural constituent of ribosomes is upregulated in MF and cytosol was abundant in CC between Δ*rfaG* and *E. coli* BW25113 strains (Figure 6b). These results indicate that *rfaG* is not directly involved in outer membrane and OMV synthesis, and possibly acts in other pathways owing to its glycosyltransferase activity [45]. However, the Δ*rfaL* strain versus *E. coli* BW25113 strain data showed substantial downregulation of the structural constituent of the ribosome, translation, and cytosolic large ribosomal subunit (Figure 6c). *RfaL* is known to encode a component of the O ligase, which transfers the completed O-antigen from the ACL to the core of a suitable LPS acceptor. Furthermore, it is involved in core modification, and plays a significant role in producing core heterogeneity, as well as O-antigen attachment [46]. The O-antigen is important for Gram-negative bacteria as it functions in targeting both the innate and adaptive immune systems in pathogenicity [47].

KEGG was also used to predict the pathway of glycolysis/glucogenesis since *rfaC*, *rfaG*, and *rfaL* were involved in LPS core glycosylation. The data revealed that Δ*rfaC* and Δ*rfaG* downregulate *ACP*, *ACEF*, and *ADHE* genes (Figure 7a). These results were likely attributable to the roles of *ACP*, *ACEF*, and *ADHE* in the transfer of acyl fatty acid during phospholipid synthesis, pyruvate dehydrogenase, and aldehyde dehydrogenase, respectively [48,49,50]. These proteins are possibly involved in LPS construction, which is implicated in the regulation of *rfaC*, *rfaG*, and *rfaL* genes. While the misfolded protein produced by a truncated set of genes will be retained in the ER and processed for ER-related degradation [51], the deletion of *rfa* genes may lead to the further interference of the membrane’s subsequent function [50]. Moreover, glycosylation deficiencies in the LPS protein production also disrupt protein localization in ER, which affects the functional membrane of protein [52]. In addition, the KEGG prediction showed high expression of PFKA, GAPA, and PYKA in *E. coli* by *rfaL* gene knockout (Figure 7b). PFKA, GAPA, and PYKA are involved in fructose phosphorylation, pyruvate formation, and GAPDH production (glycolysis pathway), respectively [53,54,55]. From these results, it is suggested that *rfaL* gene is not involved in energy production because of its role in *E. coli* LPS synthesis.

Regarding the involvement of *rfaC*, *rfaG*, and *rfaL* genes in *E. coli* LPS synthesis, a recent study mentioned that the small amount of clinically relevant Gram-negative human pathogen bacterial inoculum may cause bacteremia and eventually lead to death. *E. coli* infection in burn wound has been shown to lead to bacteremia at 24 to 48 h and death after 3 to 4 days [56]. This finding was also supported by Crompton et al., who found that in the early phase of healing, wounds treated by LPS exhibited apoptosis and reduction in local proliferation, thereby showing that contact between LPS and the wound delayed the healing process [57]. In addition, some previous studies mentioned links between glycolysis and immune system response. OMV LPS was capable of inducing macrophage metabolism shift from oxidative phosphorylation (OXPHOS) to glycolysis, which was supported by decreased mitochondrial oxygen consumption and reduced respiration activity, as well as increased mitochondrial reactive oxygen species production [58]. Thus, LPS-induced glycolysis at the wound site heightened infection by inhibiting dendritic cell maturation and inducing inflammasome activation [59,60].

Considering bacterial involvement in infection, biofilm formation could lead to further wound chronicity and delayed healing [61,62]. A previous study reported that absence of the genes responsible for generating OMV LPS, such as *rfaC*, leads to an increase in biofilm production and bacterial pathogenicity [21]. Biofilm formation may inhibit wound healing owing to a 10-fold increase in interleukin-1β (IL-1β), interleukin-6 (IL-6), and matrix metalloprotease-10 (MMP-10) expression, indicative of persistent inflammatory response and delayed healing process [63,64]. Furthermore, chronic wound biofilms can be highly tolerant and resistant to antibiotics owing to the formation of a shield to protect bacteria from the phagocytic activity of invading polymorphonuclear neutrophils (PMNs). *rfaG* gene mutation also resulted in OMV destabilization due to reduced core phosphorylation and LPS length [65,66]. This mutation also leads to a L-glycero-α-D-manno heptose (heptose) deficit that impairs the growth of *E. coli* [67]. Furthermore, due to the function of the *rfaL* gene in O-antigen biosynthesis, mutation of *rfaL* gene interferes with the transfer process of polymerized O-antigen to the lipid A core to form LPS [68]. Moreover, O-glycosylation-defective Gram-negative bacteria did not show any growth deficiency, but a tremendously diminished capacity to generate biofilm, thereby reducing its resistance against antibiotics, and affecting its survival ability in the host [69]. Hence, by deleting *rfa* genes in *E. coli*, we identified their association with OMV LPS biosynthesis, O-antigen glycosylation and biofilm formation ability. These findings can provide excellent targets for the identification of *rfa* gene inhibitors for the development of new antibiotics to enhance the wound healing process.

## 5. Conclusions

This study concludes that the OMV defects of constituent proteins RfaC, RfaG, and RfaL were upregulated, and downregulated important cellular proteins, and that they may play important roles in the glycolysis/glucogenesis pathway in *E. coli*. Hence, all findings in this study highlight that *rfaG*, *rfaC*, and *rfaL* genes are responsible for LPS synthesis as the component of OMV. Our findings also suggest that OMV contact with the injury area may inhibit the wound healing process by hindering the inflammatory response at the wound site.

## Figures and Tables

**Table 1:** *Escherichia coli* strains used in this study.

Strains	Characteristics
*E. coli* BW25113	*E. coli* BW25113 wild-type (WT) isolated from human gut microflora
*E. coli BW25113-*Δ*rfaC*	*E. coli* BW25113 lacking *rfaC* gene
*E. coli BW25113-*Δ*rfaG*	*E. coli* BW25113 lacking *rfaG* gene
*E. coli BW25113-*Δ*rfaL*	*E. coli* BW25113 lacking *rfaL* gene

**Table 2:** Unique and shared genes between Δ*rfaC*/BW25113: Δ*rfaG*/BW25113: Δ*rfaL*/BW25113.

Shared Genes			Unique Genes		
Names	Total	Elements	Names	Total	Elements
Δ*rfaC*/BW25113: Δ*rfaG*/BW25113: Δ*rfaL*/BW25113	3	*rpsT*	Δ*rfaC*/BW25113	15	*yfaZ*
		*galF*			*rpsD*
		*rpmB*			*ftnA*
Δ*rfaC*/BW25113: Δ*rfaG*/BW25113	12	*groL*			*glnA*
		*rpsM*			*htpG*
		*ygdI*			*ompF*
		*rpmA*			*rplC*
		*rpsH*			*fecA*
		*adhE*			*tufa*
		*cpoB*			*fepA*
		*rpIT*			*rplB*
		*aceF*			*lptD*
		*tig*			*yedD*
		*lepA*			*pflB*
		*agp*			*RpoC*
Δ*rfaC*/BW25113: Δ*rfaL*/BW25113	3	*rpmC*	Δ*rfaG*/BW25113	10	*rpsF*
		*clpX*			*Lpp*
		*ydiY*			*fimH*
Δ*rfaG*/BW25113: Δ*rfaL*/BW25113	3	*grpE*			*lpdA*
		*pspA*			*yajG*
		*tnaA*			*potD*
					*rplI*
					*mlaC*
					*accA*
					*rplK*
			Δ*rfaL*/BW25113	15	*gapA*
					*fliC*
					*feoB*
					*flgL*
					*gatY*
					*rplP*
					*pykA*
					*Crp*
					*pfkA*
					*aceA*
					*fliD*
					*fabB*
					*rplM*
					*Cra*

**Table 3:** Differentially expressed genes related with OMVs of genes in *E. coli* BW25113, rfaC knockout (*ΔrfaC*), *rfaG* knockout (*ΔrfaG*), and *rfaL* knockout (*ΔrfaL*) strains.

Gene	Log2 Ratio			Δ*rfaC*/BW25113		Δ*rfaG*/BW25113		Δ*rfaL*/BW25113	
	Δ*rfaC*/BW25113	Δ*rfaG*/BW25113	Δ*rfaL*/BW25113	Normalized Ratio	Trend	Normalized Ratio	Trend	Normalized Ratio	Trend
*ygdI*	2.634	2.419	−0.748	6.205	upregulated	5.347	upregulated	0.595	-
*htpG*	2.330	0.647	−0.231	5.028	upregulated	1.566	-	0.852	-
*fepA*	2.203	1.322	0.651	4.604	upregulated	2.501	-	1.571	-
*galF*	1.792	1.588	1.317	3.463	upregulated	3.006	upregulated	2.492	upregulated
*clpX*	1.788	1.270	1.543	3.453	upregulated	2.412	-	2.914	upregulated
*tufA*	1.761	1.305	0.702	3.388	upregulated	2.472	-	1.627	-
*tig*	1.690	2.258	0.461	3.226	upregulated	4.785	upregulated	1.376	-
*rplC*	1.676	1.312	−0.172	3.196	upregulated	2.484	-	0.887	-
*pflB*	1.672	0.310	0.040	3.187	upregulated	1.240	-	1.028	-
*yedD*	1.582	1.015	−0.271	2.995	upregulated	2.021	-	0.829	-
*rpsH*	1.539	1.921	0.136	2.905	upregulated	3.787	upregulated	1.099	-
*rpmA*	1.534	2.610	0.165	2.897	upregulated	6.104	upregulated	1.121	-
*lepA*	1.534	2.212	0.645	2.896	upregulated	4.634	upregulated	1.563	-
*accA*	1.413	1.829	0.910	2.663	-	3.552	upregulated	1.878	-
*rplK*	1.399	1.600	0.279	2.638	-	3.031	upregulated	1.213	-
*fabB*	1.146	1.377	1.164	2.214	-	2.597	-	2.241	upregulated
*grpE*	1.049	1.572	1.461	2.069	-	2.973	upregulated	2.754	upregulated
*mlaC*	0.950	−2.114	−0.324	1.932	-	0.231	downregulated	0.799	-
*fimH*	0.944	−1.798	−0.016	1.924	-	0.288	downregulated	0.989	-
*tnaA*	0.879	1.925	1.418	1.839	-	3.798	upregulated	2.672	upregulated
*rplI*	0.737	1.633	−0.251	1.666	-	3.101	upregulated	0.840	-
*cra*	0.544	1.199	1.457	1.458	-	2.296	-	2.745	upregulated
*gapA*	0.454	−0.106	1.372	1.370	-	0.929	-	2.589	upregulated
*potD*	0.390	−3.029	−0.621	1.311	-	0.123	downregulated	0.650	-
*rpsF*	0.386	1.523	−0.258	1.307	-	2.874	upregulated	0.836	-
*pfkA*	0.258	0.773	1.444	1.196	-	1.709	-	2.721	upregulated
*gatY*	−0.020	−1.517	1.487	0.986	-	0.349	-	2.803	upregulated
*crp*	−0.090	−0.112	1.310	0.940	-	0.925	-	2.479	upregulated
*yajG*	−0.140	−2.288	−0.815	0.907	-	0.205	downregulated	0.569	-
*aceA*	−0.146	0.561	1.231	0.904	-	1.476	-	2.347	upregulated
*fliD*	−0.148	0.238	2.531	0.902	-	1.180	-	5.778	upregulated
*lpp*	−0.150	1.700	−0.410	0.901	-	3.249	upregulated	0.753	-
*fliC*	−0.239	0.138	2.108	0.848	-	1.100	-	4.311	upregulated
*feoB*	−0.347	0.505	1.262	0.786	-	1.419	-	2.398	upregulated
*rplP*	−0.421	−1.032	−1.515	0.747	-	0.489	-	0.350	downregulated
*rplV*	−0.452	0.958	−1.197	0.731	-	1.942	-	0.436	downregulated
*flgL*	−0.502	0.170	1.231	0.706	-	1.125	-	2.346	upregulated
*pykA*	−0.538	0.937	1.210	0.689	-	1.914	-	2.313	upregulated
*rplM*	−0.711	−0.484	−1.197	0.611	-	0.715	-	0.436	downregulated
*lpdA*	−1.115	−1.944	−0.019	0.462	-	0.260	downregulated	0.987	-
*pspA*	−1.453	−1.747	−1.043	0.365	-	0.298	downregulated	0.485	downregulated
*cpoB*	−1.649	−1.615	−0.779	0.319	downregulated	0.326	downregulated	0.583	-
*agp*	−1.651	−1.890	−0.814	0.319	downregulated	0.270	downregulated	0.569	-
*rplB*	−1.695	−1.150	−0.257	0.309	downregulated	0.451	-	0.837	-
*rpsD*	−1.711	−1.532	−0.254	0.305	downregulated	0.346	-	0.838	-
*lptD*	−1.805	−1.331	−0.213	0.286	downregulated	0.398	-	0.863	-
*ydiY*	−1.835	−1.552	−1.103	0.280	downregulated	0.341	-	0.466	downregulated
*yfaZ*	−1.859	−0.389	−0.687	0.276	downregulated	0.764	-	0.621	-
*rpoC*	−1.863	−0.736	0.656	0.275	downregulated	0.601	-	1.576	-
*rpmC*	−1.905	−0.869	−1.082	0.267	downregulated	0.548	-	0.472	downregulated
*rplT*	−1.922	−1.968	0.377	0.264	downregulated	0.256	downregulated	1.299	-
*glnA*	−1.976	−0.772	0.150	0.254	downregulated	0.586	-	1.110	-
*rpsM*	−1.999	−2.230	−0.284	0.250	downregulated	0.213	downregulated	0.822	-
*fecA*	−2.064	−1.346	0.818	0.239	downregulated	0.393	-	1.763	-
*rpsT*	−2.124	−1.986	−1.968	0.229	downregulated	0.253	downregulated	0.256	downregulated
*rpmB*	−2.181	−3.315	−2.844	0.220	downregulated	0.100	downregulated	0.139	downregulated
*aceF*	−2.289	−2.137	−0.014	0.205	downregulated	0.227	downregulated	0.991	-
*ompF*	−2.563	−1.045	−0.419	0.169	downregulated	0.485	-	0.748	-
*adhE*	−2.721	−2.274	−0.703	0.152	downregulated	0.207	downregulated	0.614	-
*ftnA*	−2.772	−1.436	−0.106	0.146	downregulated	0.370	-	0.929	-
*groL*	−3.092	−2.541	−0.119	0.117	downregulated	0.172	downregulated	0.921	-

## Data Availability

Data are available upon request.
